# Overcoming lung cancer immunotherapy resistance by combining nontoxic variants of IL-12 and IL-2

**DOI:** 10.1172/jci.insight.172728

**Published:** 2023-10-09

**Authors:** Brendan L. Horton, Alicia D. D’Souza, Maria Zagorulya, Chloe V. McCreery, Gita C. Abhiraman, Lora Picton, Allison Sheen, Yash Agarwal, Noor Momin, K. Dane Wittrup, Forest M. White, K. Christopher Garcia, Stefani Spranger

**Affiliations:** 1 Koch Institute for Integrative Cancer Research at Massachusetts Institute of Technology (MIT), Cambridge, Massachusetts, USA.; 2 MIT-Harvard Health Sciences and Technology, Cambridge, Massachusetts, USA.; 3 Department of Biology, MIT, Cambridge, Massachusetts, USA.; 4 Program in Immunology,; 5 Department of Molecular and Cellular Physiology, and; 6 Department of Structural Biology, Stanford University School of Medicine, Stanford, California, USA.; 7 Department of Biological Engineering and; 8 Department of Chemical Engineering, MIT, Cambridge, Massachusetts, USA.; 9 Howard Hughes Medical Institute, Stanford University School of Medicine, Stanford, California, USA.

**Keywords:** Immunology, Cytokines, Immunotherapy, T cells

## Abstract

Engineered cytokine–based approaches for immunotherapy of cancer are poised to enter the clinic, with IL-12 being at the forefront. However, little is known about potential mechanisms of resistance to cytokine therapies. We found that orthotopic murine lung tumors were resistant to systemically delivered IL-12 fused to murine serum albumin (MSA, IL12-MSA) because of low IL-12 receptor (IL-12R) expression on tumor-reactive CD8+ T cells. IL2-MSA increased binding of IL12-MSA by tumor-reactive CD8+ T cells, and combined administration of IL12-MSA and IL2-MSA led to enhanced tumor-reactive CD8+ T cell effector differentiation, decreased numbers of tumor-infiltrating CD4+ regulatory T cells, and increased survival of lung tumor–bearing mice. Predictably, the combination of IL-2 and IL-12 at therapeutic doses led to significant dose-limiting toxicity. Administering IL-12 and IL-2 analogs with preferential binding to cells expressing Il12rb1 and CD25, respectively, led to a significant extension of survival in mice with lung tumors while abrogating dose-limiting toxicity. These findings suggest that IL-12 and IL-2 represent a rational approach to combination cytokine therapy whose dose-limiting toxicity can be overcome with engineered cytokine variants.

## Graphical abstract

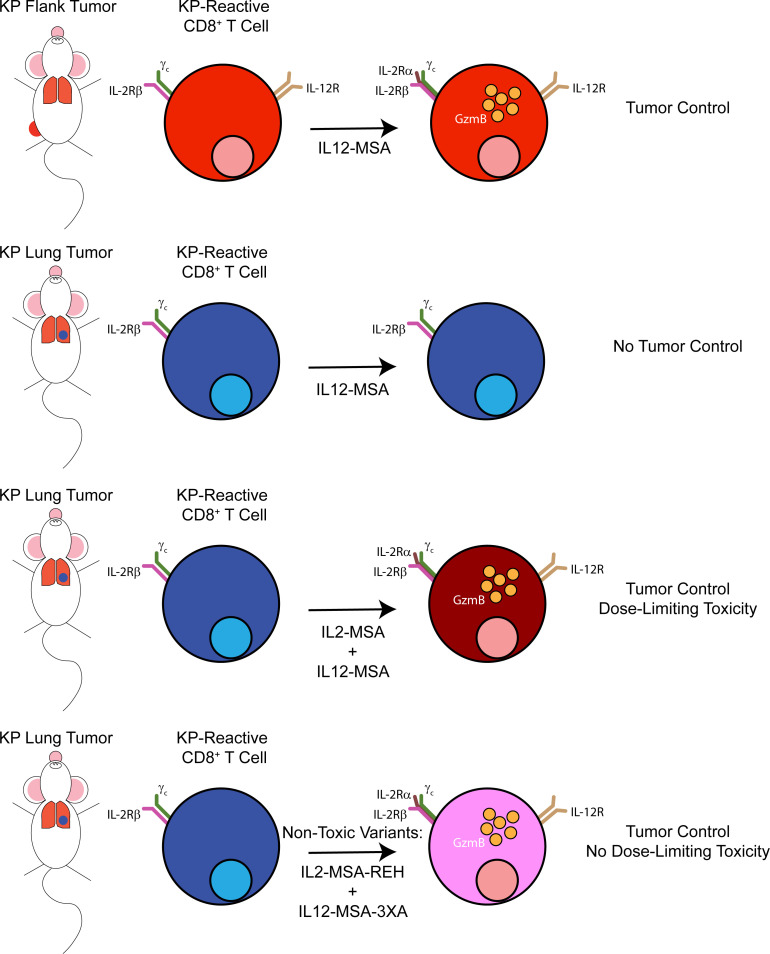

## Introduction

Immune checkpoint blockade (ICB) extends survival in non–small cell lung cancer (NSCLC) (1–4). However, most NSCLCs display either primary or secondary resistance to ICB. Primary resistance occurs when tumors lack an initial response to ICB, whereas secondary resistance occurs when tumors stop responding to ICB after an initial response (5). Current second-line therapies for ICB-resistant NSCLC have little clinical benefit (1). Therefore, improved treatment options for ICB-resistant NSCLC are needed. Preclinical studies and retrospective clinical analyses suggest that cytokine therapy can benefit a subset of patients with cancer that does not respond to ICB (6, 7). Consistent with this notion, we previously found that a transplantable murine lung tumor model (Kras^G12D^ p53^–/–^; KP) was resistant to ICB but that combined administration of IL-12 and IL-2 fused to murine serum albumin (IL12-MSA; IL2-MSA) improved control of KP lung tumors (8). In this model, combination therapy with IL12-MSA and IL2-MSA resulted in robust activation of tumor-reactive CD8^+^ T cells; however, IL12-MSA alone had no effect on CD8^+^ T cells responding to KP lung tumors. Here, we studied T cell responses to IL12-MSA alone or in combination with IL2-MSA in mice with KP lung tumors to better understand cytokine-mediated T cell activation and tumor control of ICB-resistant NSCLC.

IL-2 and IL-12 both display significant dose-limiting toxicity in humans (9–11), which has limited their clinical effect. Molecular engineering approaches have developed multiple strategies for generating cytokines with lower dose-limiting toxicity, including shielding cytokines with cleavable domains that prevent their activity until they are released by proteases in the tumor microenvironment (TME) (12), intratumorally injecting modified cytokines fused to anchoring moieties to retain them in the TME to restrict systemic toxicities (13, 14), and engineering cytokine variants with altered receptor specificities to skew cytokine activity to on-target, tumor-reactive cell populations (15, 16). While these approaches have demonstrated preclinical success, the first two restrict cytokine activity to the TME. Given the pleiotropic nature of cytokines, it is possible that cytokine-based immunotherapy could exert multiple antitumor effects across different anatomic sites. Therefore, systemic administration of cytokine variants that enables delivery to both the TME and anatomic sites outside the TME might have advantages over approaches that restrict cytokine activity to the TME alone.

In this report, we studied the efficacy of systemic IL12-MSA immunotherapy in controlling orthotopic KP lung tumors and found that KP lung tumors displayed primary resistance to systemic IL12-MSA monotherapy. KP lung tumor primary resistance to systemic IL12-MSA corresponded with low binding of IL12-MSA by tumor-reactive CD8^+^ T cells, leading to a lack of T cell activation after IL12-MSA administration. IL12-MSA binding by tumor-reactive CD8^+^ T cells was increased by IL2-MSA, and combined IL12-MSA and IL2-MSA resulted in tumor-reactive CD8^+^ T cell effector differentiation and increased lung tumor control. The proportion of CD4^+^ regulatory T cells (Tregs) in lung tumors decreased in response to either IL12-MSA alone or combined with IL2-MSA, suggesting that IL2-MSA can optimize CD8^+^ T cell responses to IL12-MSA, while IL12-MSA inhibits Treg accumulation in response to IL2-MSA. However, combined cytokine administration resulted in significant dose-limiting toxicity. To address dose-limiting toxicity, we treated tumor-bearing mice with variants of IL-2 and IL-12 that were skewed toward T cells expressing CD25 and interleukin 12 receptor subunit beta 1 (Il12rb1), respectively (15, 16). This combination resulted in a significant extension of survival after KP lung tumor challenge while eliminating dose-limiting toxicity. Together, these results show that the addition of IL-2 can overcome lung tumor primary resistance to systemic IL12-MSA monotherapy, that combined IL-2 and IL-12 orchestrate distinct effects on CD8^+^ T cells and Tregs to induce tumor control, and that the dose-limiting toxicity of this combination can be overcome by using engineered cytokine variants.

## Results

### IL12-MSA does not induce tumor control or CD8^+^ T cell activation in KP lung tumor–bearing mice.

**Figure 1:**
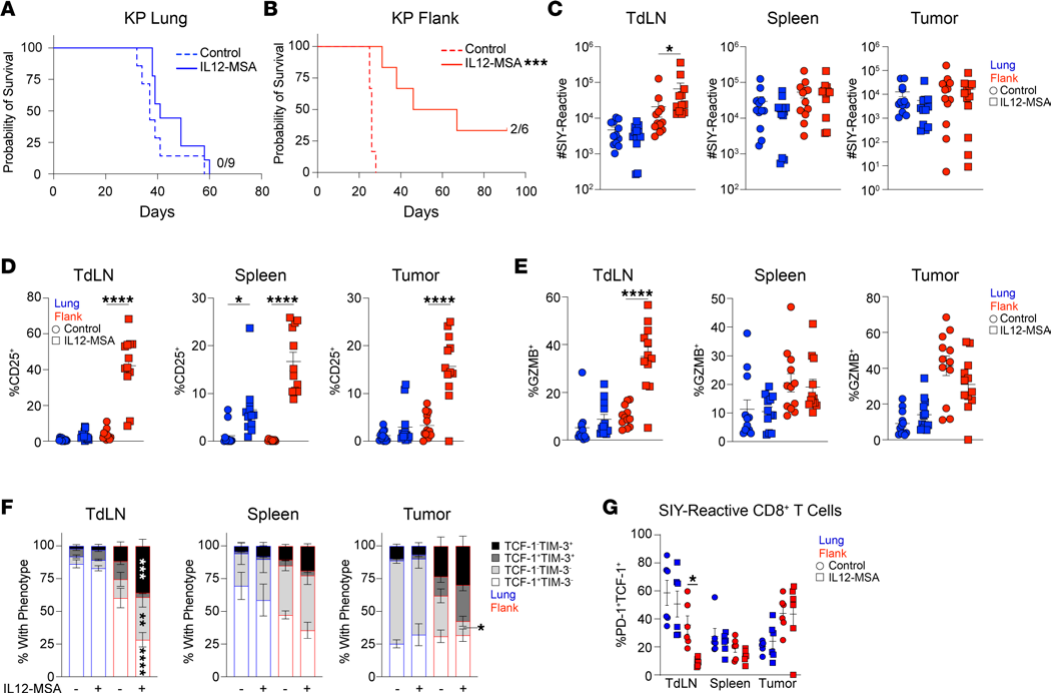
KP lung tumors are resistant to IL12-MSA monotherapy. (**A** and **B**) Mice were inoculated with KP lung or flank tumors and treated intravenously with IL12-MSA on days 7 and 14 of tumor growth. (**A**) Survival of mice with KP lung tumors, control *n* = 7, IL12-MSA *n* = 9, pooled data from 2 independent experiments. Log-rank test. (**B**) Survival of mice with KP flank tumors, control *n* = 6, IL12-MSA *n* = 6, pooled data from 2 independent experiments. Log-rank test. (**C**–**F**) Mice were inoculated with KP.SIY lung or flank tumors, treated intravenously with IL12-MSA on day 7, and analyzed on day 10 of tumor growth. (**C**) Absolute number of SIY-reactive CD8^+^ T cells from TdLN, spleen, or tumor. (**D**) CD25 expression by SIY-reactive CD8^+^ T cells from TdLN, spleen, or tumor. (**E**) GzmB expression by SIY-reactive CD8^+^ T cells from TdLN, spleen, or tumor. (**C**–**E**) *n* = 12, pooled data from 4 independent experiments, 1-way ANOVA. (**F**) TCF-1 and TIM-3 expression by SIY-reactive CD8^+^ T cells from TdLN, spleen, or tumor. (**G**) The percentage of SIY-reactive CD8^+^ T cells that are PD-1^+^TCF-1^+^. (**F** and **G**) *n* = 6, pooled data from 2 independent experiments, 2-way ANOVA. Comparisons in **F** are between control and IL12-MSA–treated samples. *P < 0.05, **P < 0.01, ***P < 0.001, ****P < 0.0001. TdLN, tumor-draining lymph node.

We previously found that combined administration of IL12-MSA and IL2-MSA could drive the effector differentiation of dysfunctional, tumor-reactive CD8^+^ T cells and extend the survival of mice with KP lung tumors (8). IL12-MSA alone, however, had little effect on the phenotype of adoptively transferred tumor-reactive CD8^+^ T cells in this model. IL12-MSA improves tumor control of multiple flank tumor models in a CD8^+^ T cell–dependent manner (13, 14). Therefore, we compared the efficacy of IL12-MSA in extending the survival of mice with either orthotopic KP lung tumors or subcutaneous KP flank tumors (Figure 1A). Strikingly, mice with KP lung tumors showed no survival benefit from IL12-MSA, while mice with KP flank tumors had significantly extended survival, and 2/6 mice with KP flank tumors completely rejected their tumors after receiving IL12-MSA (Figure 1B).

To study the endogenous tumor-reactive CD8^+^ T cell response to IL12-MSA, we utilized a KP cell line expressing the model antigen SIY (KP.SIY) (8), which is presented to CD8^+^ T cells on the MHC class I allele H2-K^b^. We compared SIY-reactive CD8^+^ T cell responses on day 10 following IL12-MSA treatment on day 7 in mice with either KP.SIY lung or flank tumors. Despite systemic administration, IL12-MSA led to the accumulation of SIY-reactive CD8^+^ T cells only in the KP.SIY flank TdLN (Figure 1C; for flow cytometry gating strategy and example plots, see Supplemental Figures 1 and 2; supplemental material available online with this article; https://doi.org/10.1172/jci.insight.172728DS1). This observation suggests that IL-12 acts predominantly on CD8^+^ T cells during priming, consistent with initial reports describing the functions of IL-12 (17–19). We next investigated aspects of CD8^+^ T cell differentiation known to be regulated by IL-12, including expression of CD25 and Granzyme B (GzmB) (20, 21). IL12-MSA upregulated CD25 expression in TdLNs, spleens, and tumors of KP.SIY flank tumor–bearing mice and spleens of KP.SIY lung tumor–bearing mice (Figure 1D), while GzmB levels were only increased in the TdLNs of KP.SIY flank tumor–bearing mice (Figure 1E). Given that IL-12 is reported to suppress expression of the transcription factor T cell factor 1 (TCF-1) in CD8^+^ T cells (22), we tested the expression of TCF-1 and T cell immunoglobulin mucin domain-containing protein 3 (TIM-3), which mark progenitor exhausted and effector-like exhausted T cell subsets, respectively (23, 24), in SIY-reactive CD8^+^ T cells after IL12-MSA treatment. IL12-MSA led to a decrease in the proportion of TCF-1–expressing, SIY-reactive CD8^+^ T cells and a concomitant increase in the proportion of TIM-3–expressing, SIY-reactive CD8^+^ T cells in the TdLNs of KP.SIY flank tumor–bearing mice (Figure 1F), indicating that IL12-MSA leads to the expansion of effector-like CD8^+^ T cells. To further determine the effect of IL12-MSA on bona fide progenitor exhausted CD8^+^ T cells, we analyzed the frequency of programmed cell death 1–positive (PD-1^+^) TCF-1^+^ cells among SIY-reactive CD8^+^ T cells (Figure 1G). We found a significant reduction in the frequency of PD-1^+^TCF-1^+^ SIY-reactive CD8^+^ T cells in flank TdLNs, but not lung TdLNs, suggesting that only in the flank tumor setting were progenitor exhausted CD8^+^ T cells responsive to IL12-MSA. Together these results suggested that IL12-MSA drives the accumulation of cytolytic, effector-like CD8^+^ T cells in the TdLNs of KP flank tumors, while having almost no effect on SIY-reactive CD8^+^ T cells in KP lung tumor–bearing mice. SIY-reactive CD8^+^ T cell activation in the TdLNs of the KP.SIY model correlated with tumor control of KP flank tumors, suggesting that CD8^+^ T cell responses are critical for mediating tumor control.

### IL2-MSA increases IL12-MSA binding by SIY-reactive CD8^+^ T cells.

**Figure 2:**
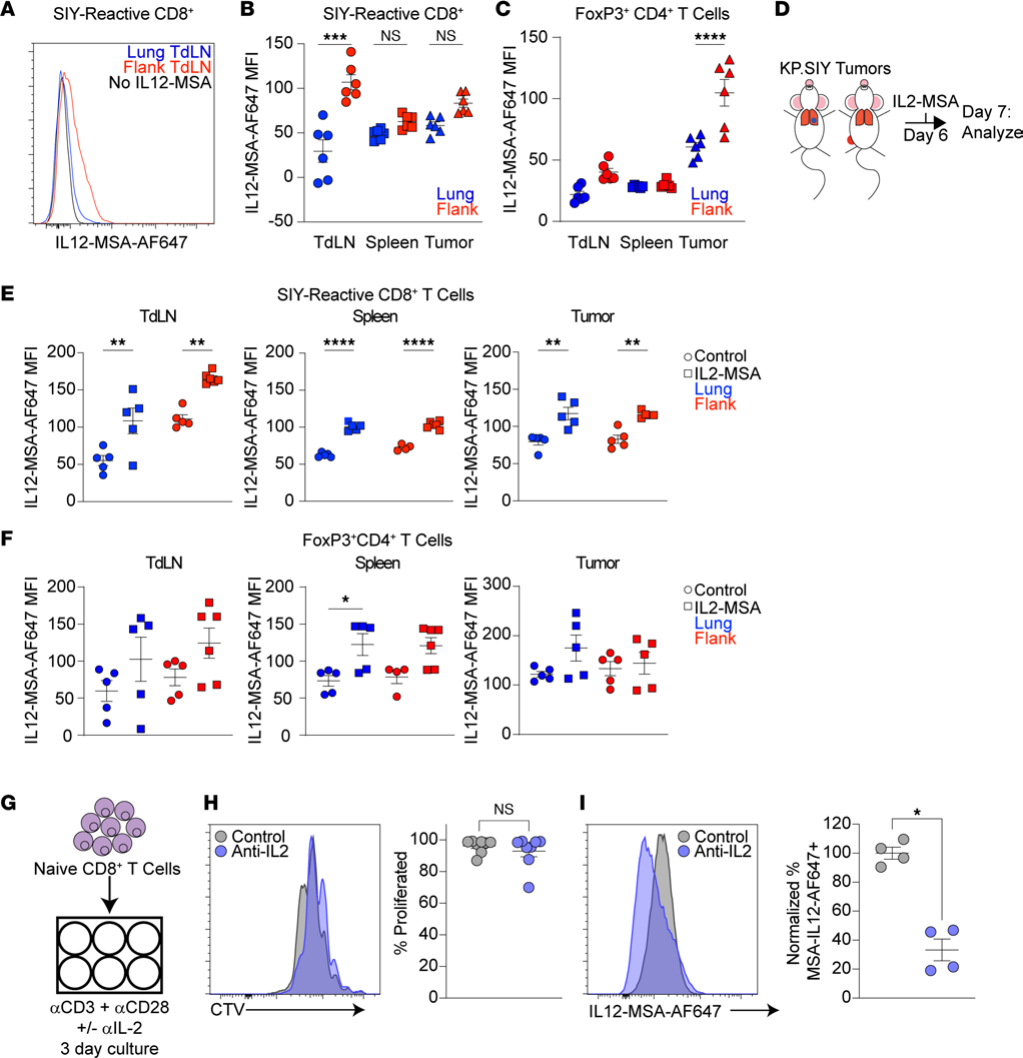
IL-2 increases CD8^+^ T cell binding of IL12-MSA. (**A**) Example histogram plot of IL12-MSA–Alexa Fluor 647 (IL12-MSA-AF647) binding by SIY-reactive CD8^+^ T cells from KP.SIY lung or flank TdLN assessed by flow cytometry. (**B** and **C**) Quantification of IL12-MSA-AF647 binding by (**B**) SIY-reactive CD8^+^ T cells and (**C**) FoxP3^+^CD4^+^ Tregs from KP.SIY lung or flank TdLN. *n* = 6, pooled data from 2 independent experiments, 1-way ANOVA. (**D**) Experimental design. KP.SIY lung or flank tumor–bearing mice were treated on day 6 of tumor growth with IL2-MSA, and T cells were analyzed ex vivo on day 7 of tumor growth (**E** and **F**). Binding of IL12-MSA-AF647 by (**E**) SIY-reactive CD8^+^ T cells and (**F**) FoxP3^+^CD4^+^ Tregs. KP.SIY lung tumors *n* = 5, all conditions. KP.SIY flank tumors TdLN control = 5, TdLN IL2-MSA *n* = 6, spleen control *n* = 4, spleen IL2-MSA *n* = 6, tumor control *n* = 5, tumor IL2-MSA *n* = 5. Pooled data from 2 independent experiments. One-way ANOVA. (**G**) Naive CD8^+^ T cells were CTV labeled and primed ex vivo with agonist anti-CD3 and anti-CD28 antibodies in the presence or absence of IL-2 neutralizing antibodies. (**H**) Example (left) and quantification (right) of primed CD8^+^ T cell proliferation, *n* = 8, pooled data from 2 independent experiments, Mann-Whitney *U* test. (**I**) Example (left) and quantification (right) of IL12-MSA binding, *n* = 4, Mann-Whitney *U* test. *P < 0.05, **P < 0.01, ***P < 0.001, ****P < 0.0001.

Our previous studies found that the quality of priming of SIY-reactive CD8^+^ T cells differs greatly between lung and flank KP.SIY TdLNs; priming of tumor-reactive CD8^+^ T cells in mediastinal TdLNs leads to significantly decreased effector T cell differentiation compared with priming in inguinal TdLNs draining flank tumors (8, 25). One consequence of this differential priming is that transcripts encoding *Il12rb1* and *Il12rb2* are decreased in SIY-reactive CD8^+^ T cells responding to KP.SIY lung tumors compared with CD8^+^ T cells responding to KP.SIY flank tumors (8). These previous results suggest that SIY-reactive CD8^+^ T cells in the KP.SIY lung tumor setting might be less responsive to IL12-MSA because of a reduced ability to bind IL12-MSA. To test this, we inoculated mice with KP.SIY tumors and used flow cytometry to assess the ability of SIY-specific CD8^+^ T cells to bind fluorescently labeled IL12-MSA ex vivo on day 7 of tumor growth. Consistent with reduced *Il12rb1* and *Il12rb2* expression by primed, SIY-reactive CD8^+^ T cells in lung TdLNs (8), SIY-reactive CD8^+^ T cells from KP.SIY lung tumor–bearing mice bound significantly less IL12-MSA than SIY-reactive CD8^+^ T cells from flank tumor–bearing mice (Figure 2, A and B). This difference in IL12-MSA binding was observed only in the TdLN, but not the spleen or tumor, consistent with our observation that SIY-reactive CD8^+^ T cell activation occurred mostly in KP.SIY flank TdLNs after IL12-MSA administration (Figure 1, C–G). We also examined fluorescently labeled IL12-MSA binding by CD4^+^FoxP3^+^ Tregs (Figure 2C) and found that in contrast with SIY-reactive CD8^+^ T cells, Tregs in tumors bound the most IL12-MSA.

IL-2 has been reported to increase the expression of IL-12R in CD4^+^ T cells (26). Therefore, we hypothesized that one mechanism of synergy between IL2-MSA and IL12-MSA might be IL2-MSA leading to increased IL-12R expression on, and increased IL12-MSA binding by, SIY-reactive CD8^+^ T cells. To test this notion, we inoculated mice with KP.SIY lung or flank tumors, treated tumor-bearing mice with IL2-MSA on day 6 of tumor growth, and analyzed the ability of SIY-reactive T cells to bind IL12-MSA ex vivo on day 7 of tumor growth (Figure 2D). Systemic administration of IL2-MSA led to significantly increased ex vivo binding of fluorescently labeled IL12-MSA by SIY-reactive CD8^+^ T cells in both the lung and flank tumor setting (Figure 2E). We also examined fluorescently labeled IL12-MSA binding by CD4^+^FoxP3^+^ Tregs after IL2-MSA administration (Figure 2F) and found in these experiments that Tregs’ IL12-MSA binding was largely unchanged by IL2-MSA. These results suggested that the synergy between IL12-MSA and IL2-MSA seen in the KP lung tumor setting is at least partly due to IL2-MSA’s ability to increase IL12-MSA binding by tumor-reactive CD8^+^ T cells.

To assess whether IL-2 signaling is required for IL12R upregulation on tumor-reactive T cells in the TdLN, we primed naive cell trace violet–labeled (CTV-labeled) CD8^+^ T cells with plate-bound CD3 and CD28 agonist antibodies in the presence or absence of neutralizing IL-2 antibodies (Figure 2G). Three days later we analyzed CD8^+^ T cell proliferation and IL12-MSA binding. We used concentrations of neutralizing IL-2 antibodies that do not inhibit CD8^+^ T cell proliferation (25), as measured by CTV dilution (Figure 2H). This level of IL-2 blockade did, however, result in significantly lower IL12-MSA binding by primed CD8^+^ T cells (Figure 2I), suggesting that sufficient IL-2 levels during CD8^+^ T cell priming are required for the expression of functional IL-12R by CD8^+^ T cells. Together these results suggest a mechanism of synergy between IL12-MSA and IL2-MSA is that IL2-MSA facilitates increased IL12-MSA binding of tumor-reactive CD8^+^ T cells by inducing IL12R upregulation. IL12-MSA can also increase CD25 expression (Figure 1D), suggesting a feed-forward loop of CD8^+^ T cell activation results from this combination.

### Combined IL12-MSA and IL2-MSA lead to rapid synergistic changes to tyrosine phosphorylation in CD8^+^ T cells.

**Figure 3:**
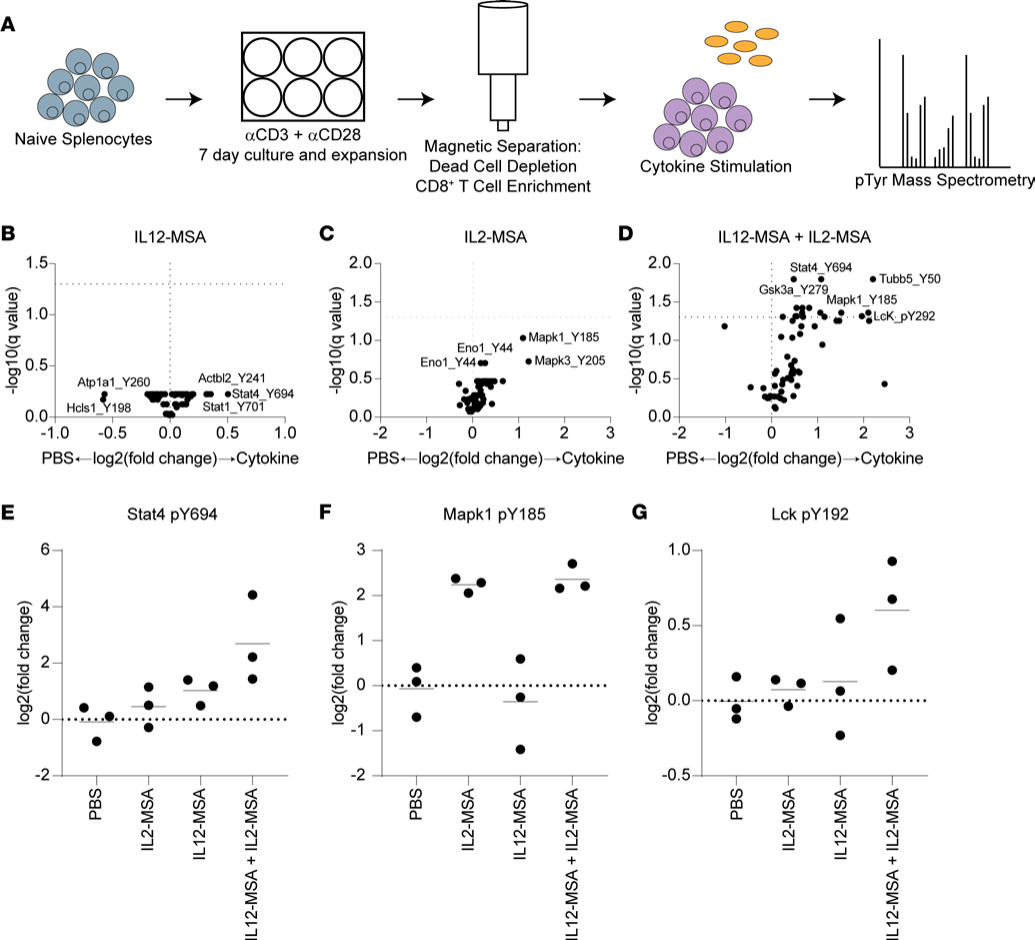
IL12-MSA and IL2-MSA induce synergistic changes to the phospho-proteome. (**A**) Experimental schematic. Naive splenocytes were activated and expanded in vitro for 7 days. CD8^+^ T cells were enriched by magnetic separation, rested, and stimulated with IL12-MSA, IL2-MSA, or the combination. Cells were lysed, and peptides were analyzed by mass spectrometry to quantify phospho-tyrosine residues. (**B**–**D**) Volcano plots quantifying changes in phospho-tyrosine sites induced by cytokine treatments. Dotted lines: *x* axis, no fold-change; *y* axis, threshold of significance. Data in **B**–**D** were generated from 3 independent biological replicates for each condition and were analyzed for significance using multiple paired *t* tests. (**E**–**G**) Plots displaying the relative abundance of specific phospho-tyrosine sites across treatment conditions.

To test whether combined IL12-MSA and IL2-MSA leads to differences in CD8^+^ T cell signaling distinct from receptor regulation, we used mass spectrometry to measure differences in tyrosine phosphorylation that occur within minutes of CD8^+^ T cell exposure to IL12-MSA and IL2-MSA. Splenocytes were activated in vitro with plate-bound agonistic anti-CD3 and anti-CD28 antibodies, and CD8^+^ T cells were isolated using a dead cell removal step followed by a CD8^+^ T cell enrichment step. Activated CD8^+^ T cells were then rested for 20 minutes, followed by a 5-minute stimulation with PBS, IL12-MSA, IL2-MSA, or combined IL12-MSA and IL2-MSA. Stimulated cells were lysed and lysates were analyzed with mass spectrometry to detect quantitative differences in phospho-tyrosine residues (Figure 3A). Neither IL12-MSA alone nor IL2-MSA alone resulted in statistically significant changes to tyrosine phosphorylation (Supplemental Figure 3 and Figure 3, B and C). The combination, however, resulted in significant increases in phosphorylation at specific tyrosine sites (Supplemental Figure 3, Supplemental Table 1, and Figure 3D), demonstrating that combined IL-12 and IL-2 can synergistically act on CD8^+^ T cell signaling in ways distinct from their individual effects. For example, Stat4-pY694 was increased by IL12-MSA alone but was further increased by combined IL12-MSA and IL2-MSA (Figure 3, D and E), despite IL2-MSA alone having no effect on Stat4-pY694. Stat4 is critical to mediating the effects of IL-12 (27, 28), suggesting that IL-2 might enhance existing IL-12 signaling by increasing Stat4 phosphorylation. Similarly, Mapk1-pY185 was increased by IL2-MSA alone but further increased by combined IL12-MSA and IL2-MSA (Figure 3, D and F), despite IL12-MSA alone having no effect on Mapk1-pY185 levels. Other significantly increased tyrosine phosphorylation sites appeared unique to the combination treatment, including Lck-pY192 (Figure 3, D and G). Combined IL12-MSA and IL2-MSA therefore appears to not only reinforce phosphorylation events that appear downstream of single-treatment conditions but also cause distinct phosphorylation events not suggested by either single treatment. Combined IL12-MSA and IL2-MSA therefore has multiple mechanisms of synergy that activate CD8^+^ T cells across different time scales, both by increasing expression of IL-2 and IL-12 receptors and through more rapidly induced changes to protein phosphorylation that support cytokine and TCR signaling.

### Combined IL12-MSA and IL2-MSA activates SIY-reactive CD8^+^ T cells while restraining Tregs in lung tumors.

**Figure 4:**
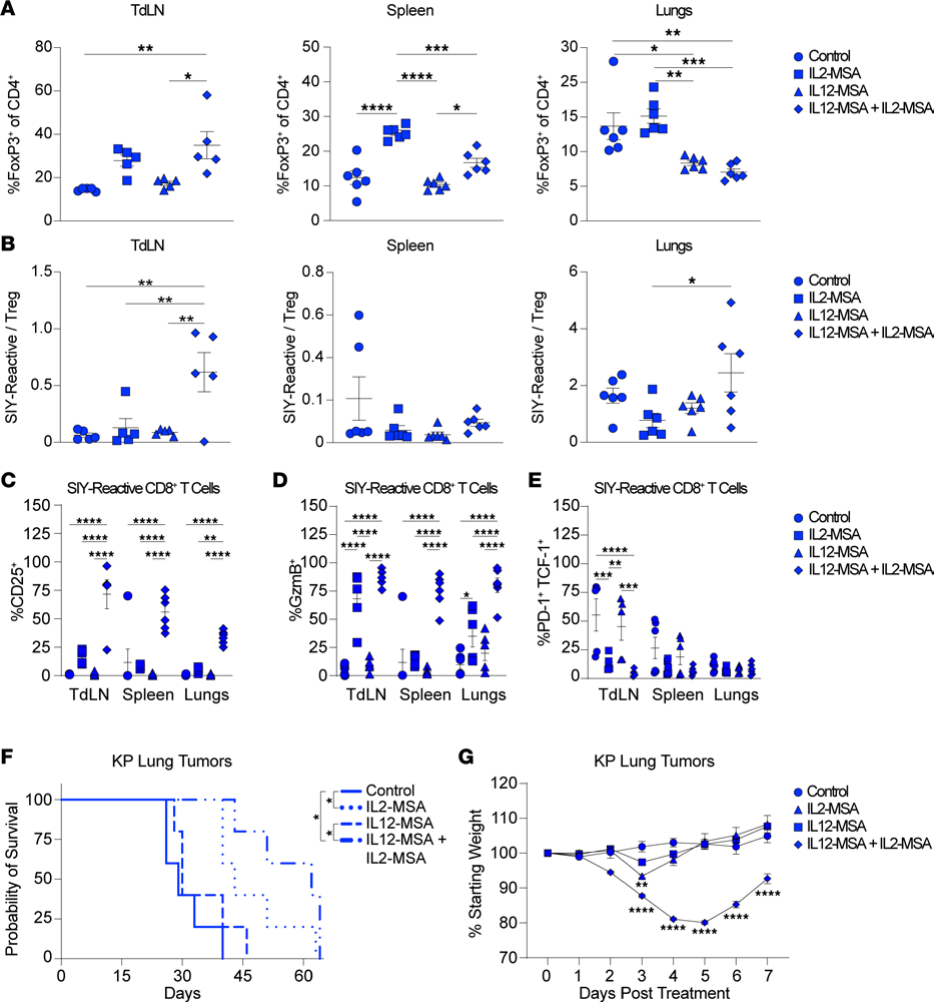
Combined IL12-MSA and IL2-MSA coordinates CD8^+^ T cell and Treg responses. (**A**–**E**) Mice were inoculated with KP.SIY lung tumors, then treated with IL12-MSA, IL2-MSA, or the combination on day 7 of tumor growth. TdLNs, spleens, and lungs were analyzed on day 10 of tumor growth. (**A**) The fraction of CD4^+^ T cells that express FoxP3. (**B**) Ratio of SIY-reactive CD8^+^ T cells to Tregs. (**C**) Percentage of SIY-reactive CD8^+^ T cells that express CD25. (**D**) Percentage of SIY-reactive CD8^+^ T cells that express GzmB. (**E**) Percentage of SIY-reactive CD8^+^ T cells that express PD-1 and TCF-1. (**A**–**E**) TdLNs *n* = 5 per condition, spleens *n* = 6 per condition, lungs *n* = 6 per condition, combined data from 2 independent experiments. (**A** and **B**) One-way ANOVA. (**C**–**E**) Two-way ANOVA. (**F** and **G**) Mice were inoculated with KP lung tumors, then treated with IL12-MSA, IL2-MSA, or the combination on days 7 and 14 of tumor growth. (**F**) Survival of KP lung tumor–bearing mice, *n* = 5 per condition, log-rank test (Mantel-Cox). Representative data from 2 experiments. (**G**) Weight loss of KP lung tumor–bearing mice treated with IL12-MSA, IL2-MSA, or the combination on day 7. Weight loss was measured from day 7 to day 14 of tumor growth. Shown statistical comparisons are with the control group, *n* = 5 per condition, 2-way ANOVA. *P < 0.05, **P < 0.01, ***P < 0.001, ****P < 0.0001.

We next determined the effects of combined IL12-MSA and IL2-MSA administration compared with either single treatment on the tumor-reactive T cell response in the context of KP.SIY lung tumors. IL-2 administration can drive both effector T cell and Treg expansion (29–33). We therefore analyzed the effects of IL12-MSA, IL2-MSA, or the combination on Tregs across anatomic sites. IL12-MSA had little effect on Treg frequency in the TdLN and spleen of KP.SIY lung tumor–bearing mice but led to a decreased Treg frequency in tumor-bearing lungs (Figure 4A). IL2-MSA led to a significantly increased Treg fraction in the spleens of KP.SIY tumor-bearing mice (Figure 4A). Interestingly, the combination of IL12-MSA and IL2-MSA led to an increased fraction of CD4^+^ T cells that were Tregs in the TdLN and spleen but a significantly decreased Treg fraction in tumor-bearing lungs (Figure 4A). Thus, IL12-MSA was able to reduce the Treg frequency in tumor-bearing lungs even in the presence of IL2-MSA, despite concomitant expansion of Tregs in secondary lymphoid tissues during combination treatment. Despite the increased percentage of Tregs in TdLNs, the SIY-reactive CD8^+^ T cell/Treg ratio increased in TdLNs during combination treatment (Figure 4B). Combination treatment also led to a significantly higher SIY-reactive CD8^+^ T cell/Treg ratio in tumor-bearing lungs compared with IL2-MSA alone (Figure 4B). Thus, while IL2-MSA can promote Treg expansion, the combination of IL12-MSA and IL2-MSA appears to favor SIY-reactive CD8^+^ T cells in the TdLN and tumor.

We next evaluated the effects of combined IL12-MSA and IL2-MSA on effector differentiation of SIY-reactive CD8^+^ T cells. CD25 expression by SIY-reactive CD8^+^ T cells was significantly upregulated by combined IL12-MSA and IL2-MSA compared with control and single-treatment conditions in the TdLNs, spleen, and lungs (Figure 4C). Both IL2-MSA and combined IL12-MSA and IL2-MSA were able to upregulate GzmB expression by SIY-reactive CD8^+^ T cells in TdLNs. However, only the combination led to significant GzmB upregulation in the spleen and lungs (Figure 4D). We also analyzed progenitor exhausted CD8^+^ T cells by assessing the PD-1^+^TCF-1^+^ percentage of SIY-reactive CD8^+^ T cells (Figure 4E). Consistent with our previous experiments (Figure 1, F and G), IL12-MSA alone did not change the fraction of SIY-reactive CD8^+^ T cells responding to KP.SIY lung tumors. However, both IL2-MSA and combined IL12-MSA with IL2-MSA decreased the fraction of SIY-reactive CD8^+^ T cells expressing both PD-1 and TCF-1 in lung TdLNs (Figure 4E). Thus, the combination of IL12-MSA and IL2-MSA was able to maximize SIY-reactive CD8^+^ T cell differentiation.

To determine the therapeutic relevance of these effects on T cell immunity, we performed survival experiments using the KP lung tumor model. We treated KP lung tumor–bearing mice with IL12-MSA, IL2-MSA, or the combination on days 7 and 14 of tumor growth and monitored mice for survival. Consistent with our original findings (Figure 1A), IL12-MSA alone had no impact on the survival of KP lung tumor–bearing mice (Figure 4F). Both IL2-MSA and the combination treatment significantly extended the survival of KP lung tumor–bearing mice, but the combination treatment led to the longest median survival (63 versus 42 days). Together, our data suggest that combined IL12-MSA and IL2-MSA promotes the activation and differentiation of SIY-reactive CD8^+^ T cells across multiple tissue sites, while restraining Tregs in tumor-bearing lungs.

To understand the dose-limiting toxicity associated with combined IL12-MSA and IL2-MSA, we measured the weights of mice for 1 week after the initial treatment on day 7 of tumor growth. IL-2 showed significant weight loss 3 days after cytokine treatment, which resolved by day 4 (Figure 4G). The combined treatment induced significant weight loss beginning 3 days posttreatment that continued through 7 days posttreatment, consistent with previous studies (14) (Figure 4G). Therefore, the combination of IL12-MSA and IL2-MSA is effective at promoting increased tumor control while inducing significant dose-limiting toxicity.

### Combined treatment with IL-12 and IL-2 variants leads to improved control of KP lung tumors without dose-limiting toxicity.

**Figure 5:**
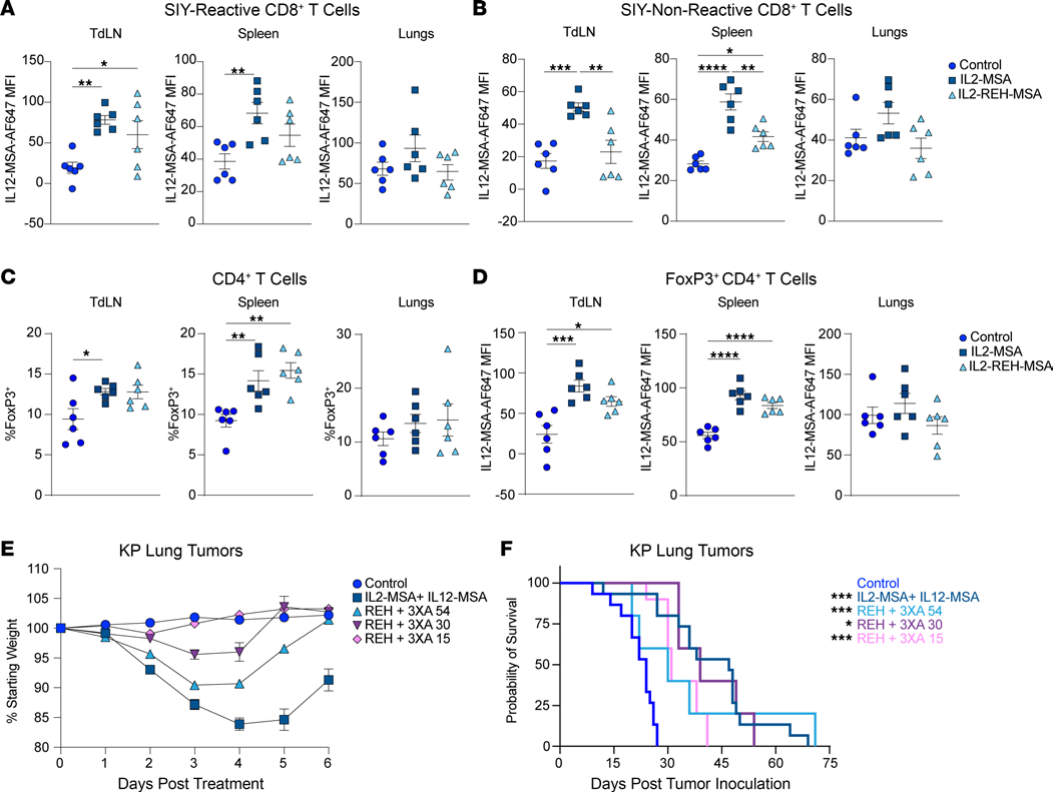
Combined administration of IL-12 and IL-2 variants extends survival of KP lung tumor–bearing mice without toxicity. (**A**–**D**) Mice were inoculated with KP.SIY lung tumors and treated with IL2-MSA or IL2-REH-MSA in vivo on day 6, and then T cells from TdLNs, spleen, and lungs were analyzed on day 7. (**A**) Binding of IL12-MSA-AF647 by SIY-reactive CD8^+^ T cells. (**B**) Binding of IL12-MSA-AF647 by bystander, SIY-nonreactive CD8^+^ T cells. (**C**) The percentage of CD4^+^ T cells that are FoxP3^+^ Tregs. (**D**) Binding of IL12-MSA-AF647 by CD4^+^FoxP3^+^ Tregs. (**A**–**D**) *n* = 6. Combined data from 2 independent experiments. One-way ANOVA. (**E** and **F**) Mice were inoculated with KP lung tumors and treated with either IL12-MSA and IL2-MSA or IL2-REH-MSA and IL12-3XA-MSA on days 7 and 14 of tumor growth. (**E**) Weight loss of KP lung tumor–bearing mice treated with either IL12-MSA and IL2-MSA or IL12-3XA-MSA and IL2-REH-MSA on day 7. Weight loss was measured from day 7 to day 14 of tumor growth. (**F**) Survival of KP lung tumor–bearing mice treated with either IL12-MSA and IL2-MSA or IL2-REH-MSA and IL12-3XA-MSA. (**E** and **F**) Control *n* = 15, IL12-MSA + IL2-MSA *n* = 15, IL2-REH-MSA + IL12-3XA-MSA 54 *n* = 5, IL2-REH-MSA + IL12-3XA-MSA 30 *n* = 5, IL2-REH-MSA + IL12-3XA-MSA 15 *n* = 10, combined data from 3 independent experiments. (**E**) Two-way ANOVA. (**F**) Log-rank test (Mantel-Cox). *P < 0.05, **P < 0.01, ***P < 0.001, ****P < 0.0001.

To address dose-limiting toxicity, we studied the effects of IL-2 (IL2-REH) and IL-12 (IL12-3XA) partial agonist analogs that are biased toward cells expressing high levels of CD25 and Il12rb1, respectively (15, 16), and fused to MSA (IL2-REH-MSA, IL12-3XA-MSA). Both cytokine variants have shown reduced dose-limiting toxicity in mice compared with the corresponding wild-type proteins (15, 16). We first asked whether IL2-REH-MSA was sufficient to increase the binding of IL12-MSA in the KP.SIY lung tumor setting. We found that indeed, one in vivo dose of IL2-REH-MSA increased the ex vivo binding of IL12-MSA by SIY-reactive CD8^+^ T cells in the TdLN (Figure 5A). We next asked whether IL2-REH-MSA reduced on-target, off-tumor effects compared with IL2-MSA. To determine whether IL2-REH-MSA could prevent the activation of non-tumor-specific CD8^+^ T cells, we compared IL12-MSA binding by SIY-nonreactive CD8^+^ T cells from mice treated with either IL2-MSA or IL2-REH-MSA. Consistent with a preference for CD25 binding, and its reduced ability to activate CD8^+^ T cells in naive mice (15), IL2-REH-MSA induced significantly less IL12-MSA binding on bystander CD8^+^ T cells than IL2-MSA (Figure 5B).

CD25 is highly expressed on CD4^+^ Tregs; therefore, we also analyzed the effects of IL2-MSA and IL2-REH-MSA on CD4^+^ Tregs. One day after administration, IL2-MSA and IL2-REH-MSA induced similar levels of CD4^+^ Treg expansion in lung TdLNs and spleens (Figure 5C). We also found that both IL2-MSA and IL2-REH-MSA increased CD4^+^ Treg binding of fluorescently labeled IL12-MSA (Figure 5D).

We next assessed whether the decreased bystander activation induced by IL2-REH-MSA would decrease the dose-limiting toxicity of combined administration of IL2-REH-MSA and IL12-3XA-MSA. We compared a dose titration of IL12-3XA-MSA while administering a constant 50 μg dose of IL2-REH-MSA. While higher doses of IL12-3XA-MSA induced weight loss similar to combined IL12-MSA and IL2-MSA, dose titration revealed that combining IL2-REH-MSA with 15 μg of IL12-3XA-MSA significantly reduced this dose-limiting toxicity, leading to no significant weight loss at any time compared to control mice (Figure 5E). We next assessed the efficacy of combined IL2-REH-MSA and IL12-3XA-MSA to extend survival in the KP lung tumor model. All dose combinations led to significantly longer survival compared with control lung tumor–bearing mice, including the 15 μg dose of IL12-3XA-MSA (Figure 5F), while IL12-3XA-MSA had no activity on its own (Supplemental Figure 4). Thus, combined, systemic administration of IL-2 and IL-12 variants improves KP lung tumor control while avoiding dose-limiting toxicity.

## Discussion

In this report, we found that primary resistance to systemic IL-12 cytokine therapy can be mediated by a lack of IL-12R expression on tumor-reactive T cells. This resistance can be overcome by combination with IL-2 therapy, and engineered cytokine variants can reduce therapy-associated dose-limiting toxicity. Specifically, we used a KP lung tumor model in which we found that single-agent, systemic IL12-MSA therapy failed to increase tumor control, while KP flank tumors were controlled by the same IL12-MSA regimen. SIY-reactive CD8^+^ T cells responding to KP.SIY lung tumors had reduced binding of IL12-MSA compared with SIY-reactive CD8^+^ T cells responding to KP.SIY flank tumors, explaining their poor activation and the lack of therapeutic efficacy of IL12-MSA in the lung tumor setting. IL2-MSA was sufficient to restore IL12-MSA binding by SIY-reactive CD8^+^ T cells, and combined IL12-MSA and IL2-MSA promoted both SIY-reactive CD8^+^ T cell effector differentiation and intratumor Treg reduction. However, this therapeutic combination led to significant dose-limiting toxicity. We found that IL-2 and IL-12 variants were able to promote increased control of KP lung tumors without dose-limiting toxicity.

This study provides many insights into the immunobiology of cytokines and how they can be harnessed therapeutically. For instance, administration of IL12-MSA alone reduced Treg frequencies in tumor-bearing lungs but was not sufficient to improve KP lung tumor control as a single-agent therapy. Previous work by our group found that tumor-reactive CD8^+^ T cell responses against KP lung tumors are dysfunctional due to Treg-mediated suppression of priming responses in the lung TdLN (8, 25). Together, these results suggest that the IL-12–mediated reduction in Treg suppression in the lung TME is insufficient to overcome the dysfunction imparted on tumor-reactive CD8^+^ T cells during priming in the lung TdLN. As previous studies have found that both global and tumor-specific Treg depletion can control established mouse models of tumors (34–37), it may therefore be of interest to future studies to compare the effects of systemic versus intratumor Treg depletion on the growth of KP lung tumors. Our group recently found that Th1-like effector state of Tregs is critical for suppression of CD8^+^ T cell priming toward lung tumors (25), and a similar Treg population was found to restrain CD8^+^ T cell responses to viral infection (38). Thus, determining the sensitivity of distinct effector Treg populations to cytokine therapy could reveal new approaches for immunotherapy.

Our current study further informs conditions that establish tumor-reactive CD8^+^ T cell dysfunction during priming. Previous studies from our group found that priming in lung TdLNs resulted in tumor-reactive CD8^+^ T cells that are activated, proliferate, and migrate into tumors but have limited cytolytic ability (8, 25). Much of this lung-specific T cell dysfunction can be attributed to Th1 effector Tregs acting in lung TdLNs to suppress dendritic cells, resulting in low costimulatory ligand expression (25), but exactly how this mediates CD8^+^ T cell dysfunction remains unclear. Previous studies found that costimulation during priming can increase IL-12R expression by T cells (39–42). We previously found that tumor-reactive CD8^+^ T cells primed in lung TdLNs express low levels of *Il12rb1* and *Il12rb2* (8), and in this report we found that these CD8^+^ T cells bind IL12-MSA poorly. Thus, low levels of costimulation provided by dendritic cells might result in low expression of IL-12R by tumor-reactive CD8^+^ T cells, leading to primary resistance to systemic IL-12 immunotherapy. It was also previously described that priming in the absence of IL-12 can result in antigen-specific CD8^+^ T cells that proliferate and expand but do not acquire cytolytic capacity (17), highlighting the requirement of IL-12 signaling for effective differentiation into cytotoxic T cells. Together, these results suggest that blunted IL-12 sensing by tumor-reactive CD8^+^ T cells undergoing priming in lung TdLNs could be a major driver of their dysfunction. Overcoming this dysfunctional state therefore requires not only additional IL-12 but also increased expression of IL-12R, which we achieved in this study by providing exogenous IL-2. As activated Tregs may also restrict IL-2 availability during priming (43), combined IL12-MSA and IL2-MSA might be able to overcome multiple inhibitory mechanisms. It may therefore be of interest to future studies to determine the contribution of costimulation versus IL-2 signaling in the endogenous upregulation of IL-12R during CD8^+^ T cell priming against lung cancer.

In addition to increasing the levels of CD25 and IL-12R, IL2-MSA and IL12-MSA synergized to alter the phospho-proteome of activated CD8^+^ T cells. Using mass spectrometry, we identified several phosphorylation sites that were significantly enriched within 5 minutes of exposure to IL2-MSA + IL12-MSA. This synergy between IL2-MSA and IL12-MSA in altering the CD8^+^ T cell phospho-proteome was more rapid than had been appreciated in previous studies of combined IL-2 and IL-12 signaling. While many of these sites may represent novel phosphorylation events downstream of combined IL-2 and IL-12 signaling, we also found phosphorylation sites in Mapk1 and Mapk3. Mapk signaling has been previously implicated in mediating the effects of combined IL-2 and IL-12 signaling (44). Thus, our mass spectroscopy data represent both an addition to and a validation of existing literature in this field.

Biomarkers that accurately predict response and resistance to therapies are becoming increasingly important for patient selection. We found that reduced IL12-MSA binding by tumor-reactive CD8^+^ T cells correlated with reduced efficacy of IL12-MSA. As novel IL-12–based therapies become approved for clinical trials (45), correlating T cell expression of IL-12R to therapeutic efficacy would determine if IL-12R expression could serve as a biomarker to aid patient selection. Low IL-12R expression could be used to predict primary resistance to IL-12–based therapies or to indicate the need for rational combinations with agents that could increase IL-12R expression, such as IL-2. Conversely, one could hypothesize that higher levels of IL-12R might predict patients with sufficient IL-2 signaling for whom additional IL-2 might have no benefit but would increase the likelihood of dose-limiting toxicity.

As systemic cytokine therapy has been previously hampered by dose-limiting toxicity (9–11), developing approaches that mitigate dose-limiting toxicity while maintaining antitumor efficacy will be critical. Approaches such as intratumor injection of cytokines are being studied to reduce systemic toxicities (13, 14). Compared with intratumor injection, systemic delivery of cytokines is simpler and less invasive, especially for tumors that are not on an exterior surface of the body and would require surgery to access or are disseminated. Other approaches to reduce systemic toxicities include engineering pro-forms of cytokines that are transformed into their active form by proteases in the TME (12). However, this approach may limit the activity of cytokines to only the TME. We found that tumor-reactive CD8^+^ T cells bind IL-12, expand, and differentiate most dramatically in the TdLNs, suggesting CD8^+^ T cells from TdLNs may be necessary for achieving maximal tumor control. Thus, we show here that IL-2 with IL-12 is a rational combination for systemic cytokine-based immunotherapy of cancer and that the dose-limiting toxicity of this combination can be overcome by protein engineering approaches.

## Methods

### Mice.

Male and female C57BL/6 mice were obtained from Taconic Biosciences (model number B6-F or B6-M) or The Jackson Laboratory (strain number 000664). All mice were housed under specific pathogen–free conditions at the Koch Institute animal facility. Mice were sex-matched and age-matched to be 6–12 weeks old at the time of experimentation. All experimental animal procedures were approved by the Committee on Animal Care at MIT.

### Tumor cell lines and tumor outgrowth studies.

The parental KP NSCLC cell line was a gift from the Jacks laboratory at MIT and validated using Dartmouse SNP analysis. Tumor cell lines were routinely screened for mycoplasma. The KP tumor line stably expressing cerulean-SIY was generated as described previously (8). Expression was periodically assessed using flow cytometry of cerulean-expressing cells. Tumor cell lines were cultured at 37°C and 5% CO_2_ in DMEM (Gibco) supplemented with 10% FBS (Atlanta Biologicals), 1% penicillin/streptomycin (Gibco), and 1× HEPES (Gibco). Tumor cells were harvested by trypsinization (Gibco) and washed 2 times with 1× PBS (Gibco). Cells were resuspended in PBS, and 2.5 × 10^5^ tumor cells were injected subcutaneously into the flanks of mice or intravenously via the tail vein. Subcutaneous tumor area measurements (calculated as length × width) were collected 2–3 times a week with calipers until the endpoint of the study. For survival studies, mice were monitored daily and euthanized when required due to tumor size or body condition.

### Tissue and tumor dissociation.

Tumor-bearing mice were injected retro-orbitally with fluorescently labeled anti-CD45 antibodies (CD45-IV) 3 minutes prior to euthanasia to differentiate tumor- and lung-infiltrating immune cells from circulating immune cells. Spleens and lymph nodes were dissected from mice and physically dissociated through a 70 μm filter (BD) to generate single-cell suspensions. Splenocyte suspensions were lysed with ACK lysis buffer (Gibco) for 3 minutes to deplete red blood cells. Subcutaneous tumors and tumor-bearing lungs were dissected from mice and collected in 1 mL DMEM (Gibco) containing DNaseI (Roche) and liberase (Roche). Tumors were shaken at 500 RPM for 30–60 minutes at 37°C. Following the digestion, tumor or lung pieces were mashed through a 70 μm filter with a 1 mL syringe plunger to generate a single-cell suspension. The dissociated cells were layered over Ficoll (GE Healthcare, now Cytiva). Cells were spun over Ficoll at 450*g* for 30 minutes with the lowest settings of acceleration and brakes. The layer at the interface of Ficoll and PBS, which contained the majority of the live immune cells, was collected and washed with PBS.

### Flow cytometry.

Cells were washed with FACS staining buffer (chilled PBS containing 1% FBS and 2 mM EDTA). Cells were resuspended in 50 μL of the antibody-containing staining buffer, plus eBioscience Fixable Viability Dye eFluor 780 (Thermo Fisher Scientific 65-0865-14) or eFluor 506 (Thermo Fisher Scientific 65-0866-14) to distinguish live and dead cells, and PE-labeled SIY-loaded MHC-I tetramers (NIH Tetramer Core Facility). Cell surface proteins were stained for 20 minutes on ice with fluorophore-conjugated antibodies at a 1:200 dilution. Cells were then washed twice and resuspended in eBioscience Fixation/Permeabilization buffer and incubated 30 minutes at room temperature. Cells were then washed twice and resuspended in staining buffer with intracellular antibodies. To obtain absolute counts of cells, Precision Count Beads (BioLegend) were added to samples according to manufacturer’s instructions. Flow cytometry sample acquisition was performed on an LSR Fortessa cytometer (BD), and the collected data were analyzed using FlowJo v10.5.3 software (Tree Star). For CD8^+^ T cell analysis, cells were pregated on forward scatter and side scatter, live, CD45^+^, CD45-IV^–^, TCRβ^+^, single cells, CD4^–^, CD8^+^. SIY-reactive CD8^+^ T cells were identified using PE-labeled, SIY-loaded MHC-I tetramers.

The following flow cytometry antibodies were used in this study: anti-CD45 clone 30-F11 conjugated to BUV395 (BD Horizon 564279) or PE-CF594 (BD Horizon 562420); anti-TCRβ clone H57-597 conjugated to BV711 (BioLegend 109207); anti-CD4 clone RM4-5 conjugated to PerCP-Cy5.5 (BioLegend 100540) or BUV737 (BD Horizon 564933); anti-CD8 clone 53.6-7 conjugated to BV605 (BioLegend 100744); anti-CD25 clone PC61 conjugated to APC-Cy7 (BioLegend 102025); anti-GzmB clone QA16A02 conjugated to AF700 (BioLegend 372221); anti–PD-1 clone RMP1-30 conjugated to PE-Cy7 (BioLegend 109110); anti–TCF-1 clone C63D9 conjugated to AF647 (Cell Signaling 6709S) or AF488 (Cell Signaling 6444S); and anti–TIM-3 clone RMT3-23 conjugated to BV421 (BioLegend 119723).

### IL12-MSA and IL2-MSA generation.

MSA-cytokine fusions were generated as previously described (14, 46). HEK293 cells (ATCC) (at 1 million cells/mL) were transfected with sterile-filtered plasmid DNA (1 mg/L cell culture) using polyethylenimine (2 mg/L cell culture) in OptiPro serum-free medium (40 mL/L cell culture) (Thermo Fisher Scientific). His-tagged proteins were isolated from HEK293 supernatant using TALON Metal Affinity Resin (Takara). Cytokine fusion proteins were then further purified by size-exclusion chromatography using a HiLoad 16/600 Superdex 200 pg column on an ÄKTA FPLC system (GE Healthcare, now Cytiva) that had been pretreated for 4 hours with 1 M NaOH to remove endotoxin and subsequently equilibrated in sterile PBS (Corning). After purification, all proteins were buffer exchanged into sterile PBS (Corning), 0.2 micron sterile-filtered (Pall Corporation), and confirmed to contain minimal endotoxin (<0.1 EU per injection) using a chromogenic LAL assay (Lonza). To confirm their molecular weights, proteins were run alongside a Novex Prestained Sharp Protein Ladder on a 4%–12% NuPAGE Bis-Tris protein gel (Life Technologies) with 1% MES running buffer. All proteins were stored at 4°C, but before therapeutic injection, cytokine fusion proteins were warmed to room temperature. Mice were treated with 50 μg IL2-MSA and 1.8 μg IL12-MSA, alone or in combination. To generate fluorescently labeled IL12-MSA, IL12-MSA was conjugated to Alexa Fluor 647 via NHS labeling (Invitrogen) (13).

### IL2-REH-MSA and IL12-3XA-MSA generation.

IL2-REH-MSA and 15 μg IL12-3XA-MSA were produced from expi293 cells (Thermo Fisher Scientific) and purified as previously described (14, 15). Mice were treated with 50 μg IL2-REH-MSA and 15 μg IL12-3XA-MSA, alone or in combination.

### In vitro CD8^+^ T cell activation, proliferation, and IL12-MSA binding.

An untreated, flat-bottom, 96-well plate was coated overnight at 4°C with 0.2 μg/mL anti-CD3 (clone 145-2C11, BD Biosciences) and 0.5 μg/mL anti-CD28 (clone 37.51, BD Biosciences) in PBS, washed with PBS, and blocked with coculture media (RPMI from Gibco containing 10% FBS from Atlanta Biologicals, 1% penicillin/streptomycin from Gibco, and 1× β-mercaptoethanol from Gibco) for at least 30 minutes at room temperature (RT). CD8^+^ T cells were isolated from the spleens of naive C57BL/6 mice using the untouched CD8^+^ T cell isolation kit (Miltenyi Biotec), following manufacturer’s instructions. Isolated CD8^+^ T cells were washed twice with PBS and stained with CTV dye (Life Technologies) following manufacturer’s instructions. Dye-labeled CD8^+^ T cells were then cultured on the anti-CD3/anti-CD28–coated plate at a density of 10^5^ cells per well in coculture media, and 2 μg/mL anti–IL-2 (using a mixture of both S4B6-1 and JES6-1A12 clones, Bio X Cell) was added at the beginning of coculture where indicated. The cells were cultured at 37°C and 5% CO_2_ for 3 days. Following coculture, cells were stained and analyzed by flow cytometry.

### In vitro T cell expansion.

Untreated, flat-bottom, 24-well plates were coated overnight at 4°C with 0.2 μg/mL anti-CD3 (clone 145-2C11, BD Biosciences) and 0.5 μg/mL anti-CD28 (clone 37.51, BD Biosciences) in PBS, washed with PBS, and blocked with coculture media for at least 30 minutes at RT. Erythrocytes were removed with ACK lysing buffer (Gibco), and splenocytes were plated at 1.2 × 10^6^ per well. Splenocytes were activated for 96 hours before being moved to 6-well plates, and fresh medium was added at a 1:1 ratio. Splenocytes were allowed to expand further 72 hours.

### Sample processing for mass spectrometry analysis.

Live CD8^+^ T cells were isolated from expanded splenocytes using a dead cell removal kit (Miltenyi Biotec 130-090-101) and a CD8^+^ T cell isolation kit (Miltenyi Biotec 130-104-075), following the manufacturer’s instructions. Approximately 12 × 10^6^ CD8^+^ T cells were resuspended in 500 μL of serum-free RPMI 1640 per condition. CD8^+^ T cells were rested for precisely 20 minutes in serum-free medium at 37°C before being stimulated with either 3 × 10^–4^ μg IL2-MSA, 3 × 10^–4^ μg IL12-MSA, or the combination. After precisely 5 minutes of stimulation, cells were lysed with a 1:1 ratio of 8 M urea. Lysates were sonicated at 30% amplitude for 10 seconds, then cleared by centrifugation at 5,000*g* for 5 minutes at 4°C, and protein concentration was measured by bicinchoninic acid assay (BCA; Pierce). Proteins were reduced with 10 mmol/L dithiothreitol for 1 hour at 56°C, alkylated with 55 mmol/L iodoacetamide for 1 hour at RT, protected from light, and diluted 4-fold with 100 mmol/L ammonium acetate, pH 8.9. Proteins were digested with sequencing-grade modified trypsin (Promega) at an enzyme/substrate ratio of 1:50 overnight at RT. Enzymatic activity was quenched by acidifying with glacial acetic acid to 10% of the final solution volume, and peptides were desalted using C18 solid-phase extraction cartridges (Sep-Pak Plus Short, Waters). Peptides were eluted with aqueous 40% acetonitrile in 0.1% acetic acid and dried using vacuum centrifugation. Protein concentration was again measured by BCA to account for variation in sample processing. Samples for TMT-labeled analyses were lyophilized in 150 mg aliquots and resuspended in 50 mmol/L HEPES (pH 8.5). TMT 6-plex (0.4 mg; Thermo Fisher Scientific) was resuspended in 15 μL of anhydrous acetonitrile and subsequently added to each sample, followed by a 1-hour incubation at RT. Reactions were quenched with the addition of hydroxylamine to a final concentration of 0.3%, pooled, dried by vacuum centrifugation, and stored at –80°C prior to analysis.

### Tyrosine phosphorylated peptide enrichment.

Lyophilized tryptic peptide aliquots were resuspended in 400 mL of immunoprecipitation (IP) buffer (100 mmol/L Tris-HCl, 0.3% NP-40, pH 7.4). The peptide mixture was incubated with 60 μL protein G agarose bead slurry (Calbiochem) conjugated to an antibody cocktail containing 24 μg 4G10 (MilliporeSigma) and 12 μg PT66 (MilliporeSigma) overnight at 4°C. Beads were washed once with IP buffer, 3 times with 100 mmol/L Tri-HCl at pH 7.4, and eluted in 2 rounds of 25 μL 0.2% TFA. Phosphopeptides were further enriched using High-Select Fe-NTA Phosphopeptide Enrichment Kit (Thermo Fisher Scientific) following manufacturer’s instructions with minor adjustments. Modifications include reducing the peptide volume initially added to the Fe-NTA column (50 μL) and reducing the elution volume to 2 rounds of 20 μL elutions. Peptide elutions were dried down using vacuum centrifugation to <2 μL total volume and resuspended in 5% acetonitrile in 0.1% formic acid for a total volume of 10 μL. Samples were loaded directly onto an in-house packed analytic capillary column (50 μm inner diameter [ID] × 10 cm) packed with 5 μm C18 beads (YMC gel, ODS-AQ, AQ12S05).

### Liquid chromatography-tandem mass spectrometry analysis.

Liquid chromatography-tandem mass spectrometry of phosphorylated tyrosine peptides was carried out on an Agilent 1260 LC coupled to an Orbitrap Exploris 480 mass spectrometer (Thermo Fisher Scientific). Peptides were separated using a 140-minute gradient with 70% acetonitrile in 0.2 mol/L acetic acid at flow rate of 0.2 mL/min with approximate split flow at 20 nL/min. The mass spectrometer was operated in data-dependent acquisition with following settings for MS1 scans: *m/z* range: 380 to 2,000; resolution: 60,000; automatic gain target (AGC) target: 3 × 10^6^; maximum injection time (maxIT): 50 ms. The top 15 abundant ions were isolated and fragmented by higher energy collision dissociation with following settings: resolution: 60,000; AGC target: 1 × 10^5^; maxIT: 250 ms; isolation width: 0.4 *m/z*, collisional energy (CE): 33%, dynamic exclusion: 50 seconds.

Crude peptide analysis was performed on a Q Exactive Plus mass spectrometer (Thermo Fisher Scientific) to correct for small variation in peptide loadings for each of the TMT channels. Approximately 30 ng of the supernatant from phosphorylated tyrosine IP was loaded onto an in-house packed pre-column (100 μm ID × 10 cm) packed with 10 μm C18 beads (YMC gel, ODS-A, AA12S11) and analyzed with a 70-minute LC gradient. MS1 scans were performed at following settings: *m/z* range: 350 to 1,800; resolution: 70,000; AGC target: 1 × 10^6^; maxIT: 100 ms. The top 10 abundant ions were isolated and fragmented with CE of 33% at a resolution of 35,000.

### Peptide identification and quantification.

Mass spectra were processed with Proteome Discoverer version 3.0 (Thermo Fisher Scientific, RRID:SCR_014477) and searched against the human SwissProt database using Mascot version 2.4 (Matrix Science, RRID:SCR_014322). MS-MS spectra were searched with mass tolerance of 10 parts per million for precursor ions and 20 milli mass unit for fragment ions. Cysteine carbamidomethylation, TMT-labeled lysine, and TMT-labeled peptide N-termini were set as fixed modifications. Oxidation of methionine and phosphorylation of serine, threonine, and tyrosine were searched as dynamic modifications. TMT reporter quantification was extracted and isotope corrected in Proteome Discoverer. Peptide spectrum matches (PSMs) were filtered according to following parameters: rank = 1, search engine rank = 1, Mascot ion score > 20. Peptides with missing values across any channel were filtered out. Phosphorylation sites were localized with ptmRS module (21) with 216.04 added as a diagnostic mass for pTyr immonium ion (22). PSMs with >95% localization probability for all phosphorylation sites were included for further analysis.

### Statistics.

Except for mass spectrometry experiments, statistics were calculated using GraphPad Prism. All data are depicted as mean ± SEM. Statistical tests were carried out as described in the figure legends. For mass spectrometry experiments, data analyses were performed in Python (version 3.6). TMT reporter ion intensities from PSMs were summed for each unique phosphopeptide. Peptide or protein quantification were normalized with relative mean values obtained from crude lysate analysis to adjust for sample loading in TMT channels. To calculate statistical significance between control and treatment groups, pairwise comparisons between control and treated sample groups were performed using 2-tailed, paired *t* test with a Benjamini-Hochberg FDR of 5% to correct for multiple testing. Unsupervised hierarchical clustering was performed on the basis of Pearson’s correlation distance metric.

### Study approval.

Animal experiments were approved by MIT’s Committee on Animal Care.

### Data availability.

Except for mass spectrometry data, all data are included in the Supporting Data Values file. The mass spectrometry proteomics data have been deposited to the ProteomeXchange Consortium via the PRIDE partner repository with the data set identifier PXD044740.

## Author contributions

BLH contributed to designing research studies, conducting experiments, acquiring data, analyzing data, and writing the manuscript. ADD contributed to designing research studies, conducting experiments, acquiring data, analyzing data, providing reagents, and writing the manuscript. MZ contributed to conducting experiments, acquiring data, and analyzing data. CVM contributed to conducting experiments and acquiring data. GCA contributed to providing reagents, designing research studies, and writing the manuscript. LP, AS, YA, NM, and KDW contributed to providing reagents. FMW contributed to providing reagents and writing the manuscript. KCG contributed to designing research studies, providing reagents, and writing the manuscript. SS contributed to designing research studies, writing the manuscript, and overseeing the study.

## Supplementary Material

Supplemental data

Supporting data values

## Version 1 09/05/2023

In-Press Preview

## Version 2 10/09/2023

Electronic publication
