# Supplementary material for: Overcoming lung cancer immunotherapy resistance by combining nontoxic variants of IL-12 and IL-2
Source: JCI Insight. 2023 Oct 9;8(19):e172728. doi: 10.1172/jci.insight.172728 (PMC10619440; doi:10.1172/jci.insight.172728)
Supplement: Supplemental data [file jciinsight-8-172728-s048.pdf]

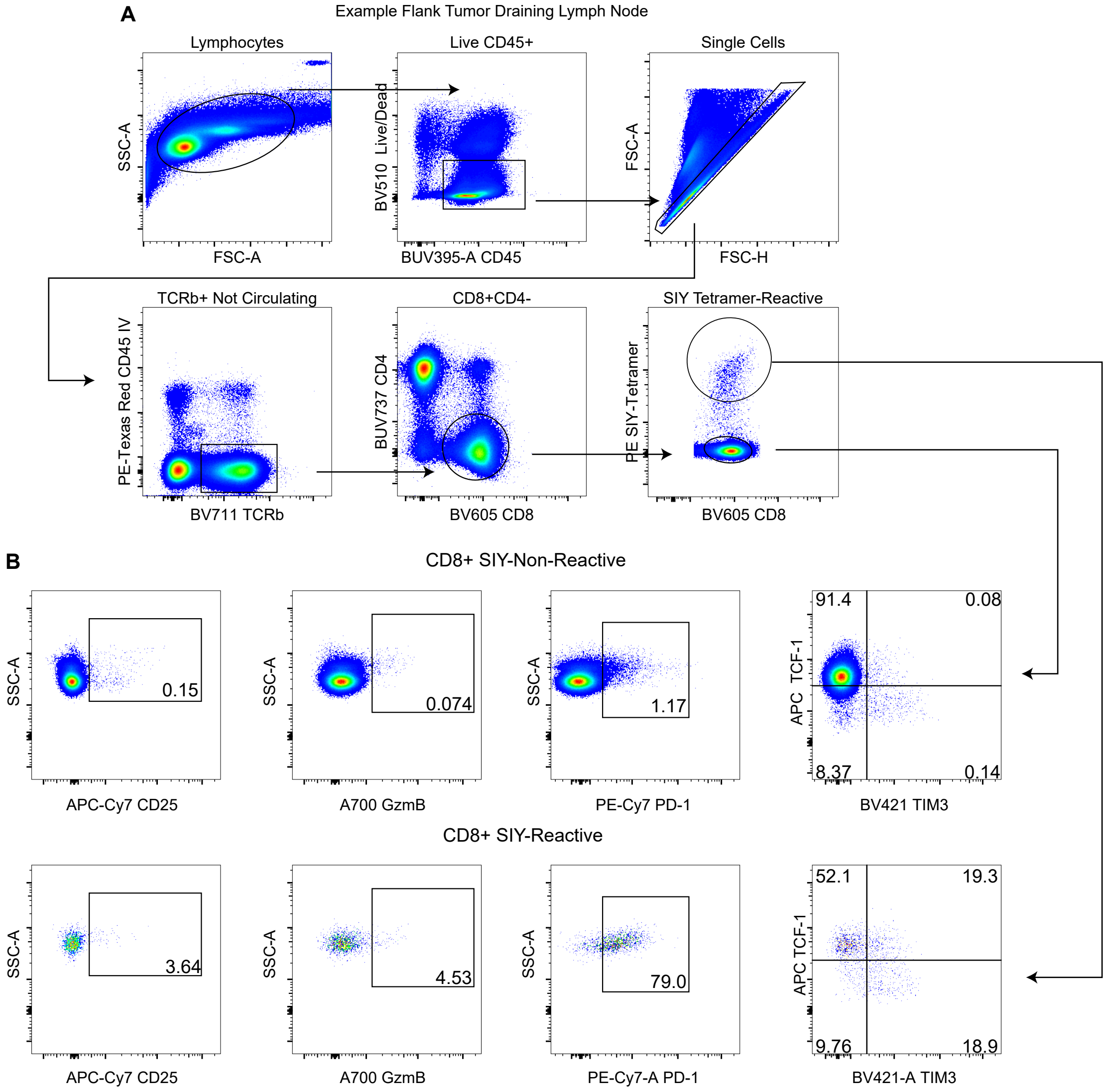
A
Example Flank Tumor Draining Lymph Node
Lymphocytes
SSC-A
FSC-A
Live CD45+
BV510 Live/Dead
BUV395-A CD45
Single Cells
FSC-A
FSC-H
TCRb+ Not Circulating
PE-Texas Red CD45 IV
BV711 TCRb
CD8+CD4-
BUV737 CD4
BV605 CD8
SIY Tetramer-Reactive
PE SIY-Tetramer
BV605 CD8
B
CD8+ SIY-Non-Reactive
SSC-A
0.15
APC-Cy7 CD25
SSC-A
0.074
A700 GzmB
SSC-A
1.17
PE-Cy7 PD-1
91.4
0.08
8.37
0.14
APC TCF-1
BV421 TIM3
CD8+ SIY-Reactive
SSC-A
3.64
APC-Cy7 CD25
SSC-A
4.53
A700 GzmB
SSC-A
79.0
PE-Cy7-A PD-1
52.1
19.3
9.76
18.9
APC TCF-1
BV421-A TIM3

Supplemental Figure 1.

(A) Representative gating strategy to identify SIY tetramer-reactive $CD8^+$ T cells. **(B)** Representative gating of CD25, GzmB, PD-1, TCF-1, and TIM-3 on SIY-reactive and SIY non-reactive $CD8^+$ T cells.

Example Tumor-Draining Lymph Nodes

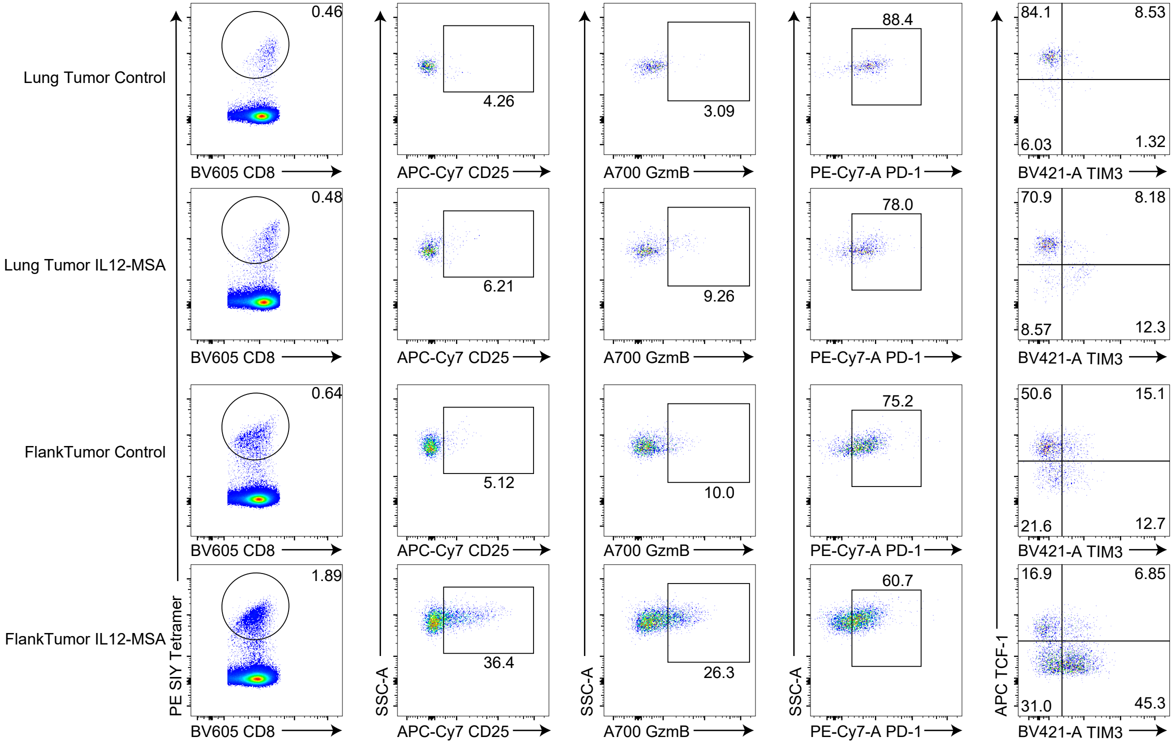

Supplemental Figure 2.

Representative gating of SIY tetramer-reactive CD8^{+} T cells and their expression of CD25, GzmB, PD-1, TCF-1, and TIM-3 across different tumor sites and treatment conditions. Images depict tumor-draining lymph nodes. The same gating strategies were used for analyzing spleens and tumors.

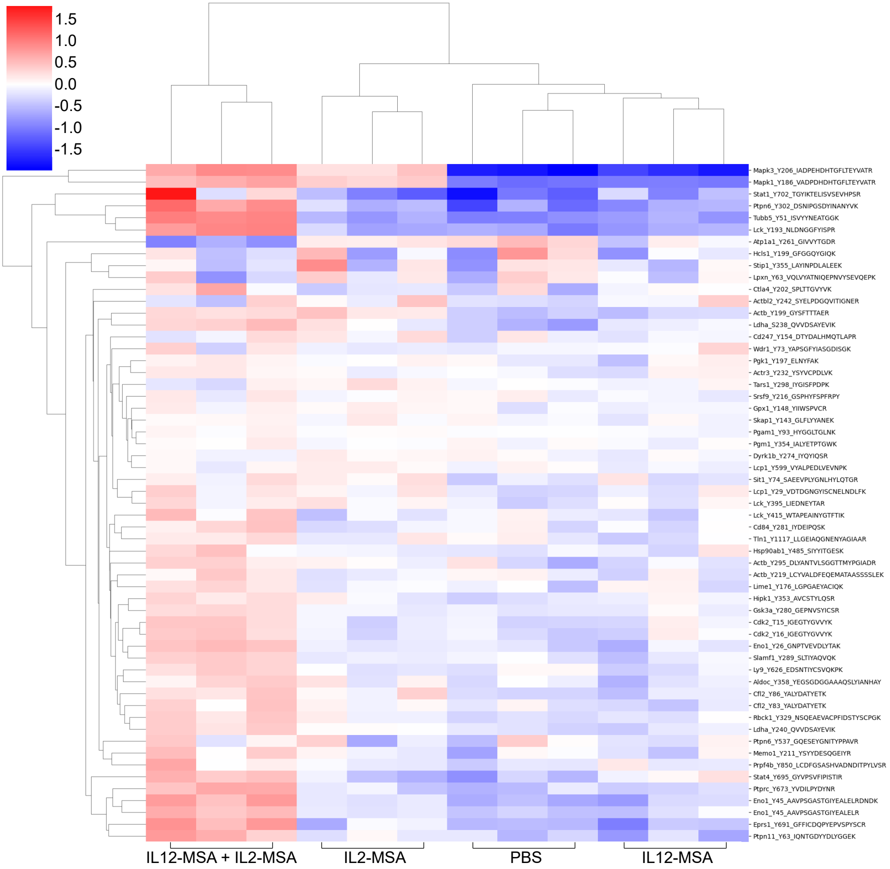
1.5
1.0
0.5
0.0
-0.5
-1.0
-1.5
Mapk3_Y206_IADPEHDHTGFLTEYVATR
Mapk1_Y186_VADPDHDHTGFLTEYVATR
Stat1_Y702_TGYIKTELISVSEVHPSR
Ptpn6_Y302_DSNIPGSDYINANYVK
Tubb5_Y51_ISVYYNEATGGK
Lck_Y193_NLDNGGFYISPR
Atp1a1_Y261_GIVVYTGDR
Hcls1_Y199_GFGGQYGIQK
Stip1_Y355_LAYINPDLALEEK
Lpxn_Y63_VQLVYATNIQEPNVYSEVQEPK
Ctla4_Y202_SPLTTGVYVK
Actbl2_Y242_SYELPDGQVITIGNER
Actb_Y199_GYSFTTTAER
Ldha_S238_QVVDSAYEVIK
Cd247_Y154_DTYDALHMQTLAPR
Wdr1_Y73_YAPSGFYIASGDISGK
Pgk1_Y197_ELNYFAK
Actr3_Y232_YSYVCPDLVK
Tars1_Y298_IYGISFPDPK
Srsf9_Y216_GSPHYFSPFRPY
Gpx1_Y148_YIIWSPVCR
Skap1_Y143_GLFLYYANEK
Pgam1_Y93_HYGGLTGLNK
Pgm1_Y354_IALYETPTGWK
Dyrk1b_Y274_IYQYIQSR
Lcp1_Y599_VYALPEDLVEVNPK
Sit1_Y74_SAEEVPLYGNLHYLQTGR
Lcp1_Y29_VDTDGNGYISCNELNDLFK
Lck_Y395_LIEDNEYTAR
Lck_Y415_WTAPEAINYGTFTIK
Cd84_Y281_IYDEIPQSK
Tln1_Y1117_LLGEIAQGNENYAGIAAR
Hsp90ab1_Y485_SIYYITGESK
Actb_Y295_DLYANTVLSGGTTMYPGIADR
Actb_Y219_LCYVALDFEQEMATAASSSSLEK
Lime1_Y176_LGPGAEYACIQK
Hipk1_Y353_AVCSTYLQSR
Gsk3a_Y280_GEPNVSYICSR
Cdk2_T15_IGEGTYGVVYK
Cdk2_Y16_IGEGTYGVVYK
Eno1_Y26_GNPTVEVDLYTAK
Slamf1_Y289_SLTIYAQVQK
Ly9_Y626_EDSNTIYCSVQKPK
Aldoc_Y358_YEGSGDGGAAAQSLYIANHAY
Cfl2_Y86_YALYDATYETK
Cfl2_Y83_YALYDATYETK
Rbck1_Y329_NSQEAEVACPFIDSTYSCPGK
Ldha_Y240_QVVDSAYEVIK
Ptpn6_Y537_GQESEYGNITYPPAVR
Memo1_Y211_YSYYDESQGEIYR
Prpf4b_Y850_LCDFGSASHVADNDITPYLVSR
Stat4_Y695_GYVPSVFIPISTIR
Ptprc_Y673_YVDILPYDYNR
Eno1_Y45_AAVPSGASTGIYEALELRDNDK
Eno1_Y45_AAVPSGASTGIYEALELR
Eprs1_Y691_GFFICDQPYEPVSPYSCR
Ptpn11_Y63_IQNTGDYYDLYGGEK
IL12-MSA + IL2-MSA
IL2-MSA
PBS
IL12-MSA

Supplemental Figure 3.

Heatmap of selected phosphorylation sites. Unsupervised hierarchical clustering was performed on the basis of the Pearson correlation distance metric.

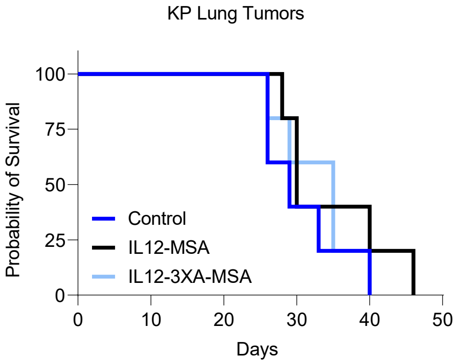
KP Lung Tumors
Probability of Survival
100
75
50
25
0
0
10
20
30
40
50
Days
Control
IL12-MSA
IL12-3XA-MSA

Supplemental Figure 4.

Kaplan-Meier plot of mice bearing KP lung tumors. Mice were treated with either IL12-MSA or IL12-3XA-MSA on days 7 and 14 post-tumor inoculation and monitored for survival. N = 5 for all conditions. Log-rank test (Mantel-Cox).

Significantly Different Phospho Sites								
	P value	Mean of IL12+IL2	Mean of PBS	Difference	SE of difference	t ratio	df	q value
Stat4_Y694	0.000581	1.654	0.5745	1.08	0.02603	41.49	2	0.015927
Gsk3a_Y279	0.000631	1.333	0.8513	0.4818	0.01211	39.79	2	0.015927
Tubb5_Y50	0.000758	2.594	0.3908	2.203	0.0607	36.29	2	0.015927
Cdk2_Y15	0.002752	1.453	0.7831	0.6703	0.03523	19.02	2	0.037617
Memo1_Y210	0.003528	1.382	0.8407	0.5417	0.03227	16.79	2	0.037617
Eno1_Y25	0.003583	1.604	0.7866	0.8169	0.04903	16.66	2	0.037617
Mapk1_Y185	0.005997	1.857	0.3432	1.514	0.1178	12.85	2	0.043623
Actb_Y198	0.006111	1.323	0.6585	0.6643	0.05217	12.73	2	0.043623
Mapk3_Y205	0.0065	2.263	0.159	2.104	0.1705	12.34	2	0.043623
Eno1_Y44	0.006924	1.718	0.6569	1.061	0.08872	11.95	2	0.043623
Lck_Y192	0.008918	2.432	0.4697	1.963	0.1866	10.52	2	0.048384
Slamf1_Y288	0.009658	1.447	0.8968	0.5498	0.05442	10.1	2	0.048384
Hipk1_Y352	0.009984	1.291	0.7696	0.5214	0.05249	9.933	2	0.048384
Cdk2_T14	0.011685	1.466	0.781	0.6846	0.07466	9.169	2	0.049173
Ptprc_Y672	0.01192	1.813	0.663	1.15	0.1267	9.077	2	0.049173
Actr3_Y231	0.012488	1.148	0.9058	0.2422	0.02732	8.864	2	0.049173

Supplemental Table 1.

Phosphorylation sites that are statistically significantly different between control and IL2-MSA + IL12-MSA treated samples.
